# Purification and Characterization of a Novel Chlorpyrifos Hydrolase from *Cladosporium cladosporioides* Hu-01

**DOI:** 10.1371/journal.pone.0038137

**Published:** 2012-06-05

**Authors:** Yan Gao, Shaohua Chen, Meiying Hu, Qiongbo Hu, Jianjun Luo, Yanan Li

**Affiliations:** 1 Key Laboratory of Pesticide and Chemical Biology, Ministry of Education, South China Agricultural University, Guangzhou, People's Republic of China; 2 Guangdong Provincial Key Laboratory of High Technology for Plant Protection, Plant Protection Research Institute, Guangdong Academy of Agricultural Sciences, Guangzhou, People's Republic of China; Missouri University of Science and Technology, United States Of America

## Abstract

Chlorpyrifos is of great environmental concern due to its widespread use in the past several decades and its potential toxic effects on human health. Thus, the degradation study of chlorpyrifos has become increasing important in recent years. A fungus capable of using chlorpyrifos as the sole carbon source was isolated from organophosphate-contaminated soil and characterized as *Cladosporium cladosporioides* Hu-01 (collection number: CCTCC M 20711). A novel chlorpyrifos hydrolase from cell extract was purified 35.6-fold to apparent homogeneity with 38.5% overall recovery by ammoniumsulfate precipitation, gel filtration chromatography and anion-exchange chromatography. It is a monomeric structure with a molecular mass of 38.3 kDa. The pI value was estimated to be 5.2. The optimal pH and temperature of the purified enzyme were 6.5 and 40°C, respectively. No cofactors were required for the chlorpyrifos-hydrolysis activity. The enzyme was strongly inhibited by Hg^2+^, Fe^3+^, DTT, *β*-mercaptoethanol and SDS, whereas slight inhibitory effects (5–10% inhibition) were observed in the presence of Mn^2+^, Zn^2+^, Cu^2+^, Mg^2+^, and EDTA. The purified enzyme hydrolyzed various organophosphorus insecticides with P-O and P-S bond. Chlorpyrifos was the preferred substrate. The *K*m and *V*max values of the enzyme for chlorpyrifos were 6.7974 μM and 2.6473 μmol·min^−1^, respectively. Both NH2-terminal sequencing and matrix-assisted laser desorption/ionization time-of-flight/time-of-flight mass spectrometer (MALDI-TOF-MS) identified an amino acid sequence MEPDGELSALTQGANS, which shared no similarity with any reported organophosphate-hydrolyzing enzymes. These results suggested that the purified enzyme was a novel hydrolase and might conceivably be developed to fulfill the practical requirements to enable its use in situ for detoxification of chlorpyrifos. Finally, this is the first described chlorpyrifos hydrolase from fungus.

## Introduction

Chlorpyrifos (*O*, *O*-diethyl *O*-(3,5,6-trichloro-2-pyridyl) phosphorothioate) is one of the most widely used organophosphorus insecticides effective against a broad spectrum of insect pests of economically important crops [1]. It is also extensively used for the control of mosquitoes, flies, termites, and various veterinary and household pests [2], [3]. Its half-life in soil is usually between 60 and 120 days, but can range from 2 weeks to over 1 year, depending on the soil type, pH, temperature, and other conditions [4], [5]. The persistent use of chlorpyrifos has led to widespread contamination of water and soils, resulting in serious damage to non-target species [6], [7]. For instance, chlorpyrifos may affect the central nervous system, the cardiovascular system, as well as the respiratory system due to its high acute toxicity [8]. Furthermore, the frequent use of chlorpyrifos in agriculture has increased the public concern on potential human health risks that may result from acute or chronic dietary exposure to chlorpyrifos residues on food [9], [10]. Therefore, it is necessary to develop remediation methods to degrade and eliminate this pollutant from environments.

Several methods have been used for removal of chlorpyrifos including chemical treatment, open-pit burning, deep ocean dumping, and incineration [11]. As a result, disposal of the contaminant following the conventional engineering approaches based on physicochemical methods is both technically and economically challenging [12]. The fate of chlorpyrifos in the environment is associated with both abiotic and biotic process, including photolysis, chemical hydrolysis and microbial degradation [7]. Microbial degradation is considered to be the primary mechanism determining the fate and behavior of chlorpyrifos [13]. Studies on microbial degradation are very important in the development of bioremediation strategies for the detoxification of chlorpyrifos and other pesticides [14], [15]. Bioremediation is a biological process that uses living organisms or their products (enzymes) to convert a harmful substance to a non-toxic substance or to return the contaminated environment to its original condition [16]. Recently, bioremediation has received increasing attention as an effective, cheap and safe approach to clean up contaminated environments [17], [18]. Several chemicals have been successfully removed from soils and aquatic environments using degrading microorganisms [19]–[23]. To date, a few chlorpyrifos-degrading bacteria including *Enterobacter* strain B-14 [24], *Alcaligenes faecalis* DSP3 [25], *Stenotrophomonas* sp. strain YC-1 [26], *Sphingomonas* sp. strain Dsp-2 [27], *Paracoccus* sp. strain TRP [7], and *Bacillus pumilus* strain C2A1 [1] have been isolated from contaminated soils, industrial wastewater, as well as polluted sediments. The only hydrolase gene (*mpd*) involved in the degradation of chlorpyrifos has been cloned after extraction from *Stenotrophomonas* sp. strain YC-1 [26] and *Sphingomonas* sp. strain Dsp-2 [27]. There are, however, rare reports about fungi strains responsible for chlorpyrifos degradation, e.g. only *Verticillium* sp. strain DSP [28] and *Acremonium* sp. strain GFRC-1 [13] isolated from contaminated soils using an enrichment culture technique. In addition, the existing papers lack the information on the genetic and enzymatic aspects involved in the degradation of chlorpyrifos by fungi. Fungi possess the biochemical and ecological capacity to degrade environmental organic chemicals, either by chemical modification or by influencing chemical bioavailability [29]. Furthermore, the ability of fungi to form extended mycelial networks, the low specificity of their catabolic enzymes and their independence from using xenobiotics as a growth substrate make fungi well suited for bioremediation processes [29]. To the best of our knowledge, this is the first report about a fungus of the genus *Cladosporium* that can degrade chlorpyrifos.

In the present study, we describe the purification and characterization of a novel chlorpyrifos hydrolase from *Cladosporium cladosporioides* Hu-01, previously isolated from the organophosphorus pesticides contaminated soils. The objective of this study was to investigate its specific role on chlorpyrifos degradation. To our knowledge, this is the first chlorpyrifos hydrolase purified to homogeneity from fungi, and further genetic studies may lead to the discovery of novel genes involved in the future.

## Materials and Methods

### Chemicals and reagents

Chlorpyrifos standard (97% purity) was obtained from Dow AgroSciences, USA. Sephacryl^TM^ S-100 (16/60), HiTrap^TM^ IEX Kit, and diethylaminoethyl cellulose (DEAE) were purchased from General Electric Company, USA. Chromatographic grade methanol were purchased from Sigma-Aldrich, USA. Sodium dodecyl sulfate (SDS) and polyacrylamide were purchased from Amresco, USA. Polyvinylidene fluoride (PVDF) membrane was purchased from Millipore, USA. All other chemicals and solvents used were analytical grade and purchased from Merck, Germany.

### Microorganism isolation and cultivation conditions

*C. cladosporioides* Hu-01, which was employed here, was isolated from the organophosphorus pesticides contaminated soils using an enrichment culture technique. The enrichment medium (Czapek-Dox) containing (in gram per litre) 30 g of sucrose, 2 g of NaNO_3_, 0.5 g of KCl, 0.5 g of MgSO_4_, 1 g of K_2_HPO_4_, 0.01 g of Fe_2_(SO_4_)_3_, 0.5 g peptone and the mineral salt medium (MSM) containing (in gram per litre) 2.0 g of (NH_4_)_2_SO_4_, 0.2 g of MgSO_4_·7H_2_O, 0.01 g of CaCl·2H_2_O, 0.001 g of FeSO_4_·7H_2_O, 1.5 g of Na_2_HPO_4_·12H_2_O, 1.5 g of KH_2_PO_4_ were used for the isolation of fungal strains. Enrichment and isolation of fungi were performed as described in detail previously [30], [31]. In brief, two gram of soil sample was transferred into a 250-mL Erlenmeyer flask containing 50 mL MSM with the addition of 50 mg·L^−1^ chlorpyrifos as the sole carbon source and incubated at 28°C for 7 days in a rotary shaker at 150 rpm. Five milliliters of the enrichment culture was transferred into 50 mL fresh enrichment medium and incubated for another 7 days. After five rounds of transfer, the final culture was serially diluted and spread on Czapek-Dox agar plates. The strain Hu-01 that could make use of chlorpyrifos as the sole carbon source to grow on the MSM was deposited in China Center for Type Culture Collection (collection number: CCTCC M 20711).

### Enzyme purification

All purification steps were carried out at 4°C, unless otherwise specified. Purification was performed by the method of Liang et al. [32] with modification.

- Preparation of crude extract. For enzyme production, the fresh MSM containing 50 mg·L^−1^ of chlorpyrifos was inoculated with *C. cladosporioides* Hu-01 viable spores. The culture was incubated at 28°C for 5 days in 500 mL-Erlenmeyer flasks containing 200 mL of medium on a rotary shaker at 150 rpm, harvested by centrifugation at 8000×*g* for 30 min at 4°C, washed twice with cold 0.05 M phosphate buffer (pH 6.5), and stored at −20°C until used later.
  - Next, 20 g of washed mycelia was resuspended in 0.05 M phosphate buffer (pH 6.5) and disrupted in an ultrasonic cell disruption system (Scientz, China). After standing at 4°C overnight, the suspension was centrifuged (12000×*g* for 10 min at 4°C) to remove the unbroken cells and cellular debris. The suspension was subjected to centrifugation at 15000×*g* for 20 min, and the resulting supernatant was used as an enzyme source for subsequent enzyme purification.
- Ammonium sulfate precipitation. The 15000×*g* supernatant was brought to 20% ammonium sulfate saturation and stirred for 30 min, the cloudy suspension was centrifuged at 15000×*g* for 20 min, and supernatant was brought to 90% ammonium sulfate saturation; after being stirred for 30 min, the pellet obtained by centrifugation at 15000×*g* for 20 min was dissolved in the smallest possible volume 0.05 M phosphate buffer (pH 6.5) and dialyzed 1000-fold against 0.05 M phosphate buffer (pH 6.5), and the supernatant was filtered through a 0.22 μm membrane (Millipore, USA) and concentrated.
- Sephacryl^TM^ S-100 (16/60) gel filtration. The concentrated enzyme solution was applied to Sephacryl^TM^ S-100 column (2.5×100 cm) preequilibrated with 0.05 M phosphate buffer (pH 6.5) using a ÄKTA Explorer 100 system (General Electric Company, USA). The column was washed at a flow rate of 0.5 mL·min^−1^ with 180 mL of the same buffer, and 5-mL fractions were collected. The fractions with high specific activity were then pooled and concentrated for further purification.
- HiTrap^TM^ IEX ion-exchange chromatography. The concentrated enzyme was placed on a DEAE Sepharose Fast Flow anion-exchange column (2.5×30 cm) that had been equilibrated with 0.05 M phosphate buffer (pH 6.5). The column was washed with 20 mL of the same buffer, and proteins were eluted with a linear gradient of NaCl solution in the range of 0 to 1.0 M in the equilibrating buffer. Fractions (5 mL) were collected every 5 min and screened for enzyme activity. Active fractions were pooled for subsequent analysis.

### Protein determination

Protein concentration was measured by the method of Bradford [33] with bovine serum albumin as standard using a spectrophotometer (Shimadzu, Japan).

### Enzyme activity determination

Pesticide hydrolase activity assays were carried out by adding 0.2 mL enzyme solution to 3 mL of 0.05 M phosphate buffer (pH 6.5) containing 50 mg·L^−1^ of chlorpyrifos and incubating for 10 min at 40°C. After that, samples were extracted, and the remaining pesticides were measured by high performance liquid chromatography (HPLC). Every treatment was performed in triplicate with inactivated enzyme as control. One unit of enzyme activity (U) was defined as the amount of enzyme that hydrolyzed 1 mmol of chlorpyrifos per minute from substrate under these conditions.

### Molecular mass and pI determination

The molecular mass of the denatured protein was investigated using SDS-PAGE (Amresco, USA). An SDS-12.5% polyacrylamide gel was prepared according to the method of Laemmli [34] with modification. Proteins were stained with Coomassie brilliant blue R-250 [35]. The molecular mass of the native protein was determined by gel filtration on the Sephacryl^TM^ S-100 (16/60). Phosphorylase b (97.2 kDa), bovine serum albumin (66.4 kDa), ovalbumin (44.3 kDa), carbonic anhydrase (29.0 kDa), trypsin inhibitor (20.1 kDa), and lysozyme (14.3 kDa) were used as the standard proteins. Isoelectric point (pI) was estimated using isoelecric focusing calibration [32], [36].

### Effects of pH and temperature on hydrolase activity

For determination of the optimal pH and temperature, the hydrolase activity was investigated by incubating the purified enzyme (0.2 mL) with 50 mg·L^−1^ chlorpyrifos as a substrate for 10 min in 0.05 M phosphate buffer. At temperature 40°C, hydrolase activity was assayed at pH values ranging from 4.0 to 10.0 in 0.5 of increment. At pH 6.5, hydrolase activity was assayed between 20°C and 60°C in 5°C of increment. The relative residual activity was measured as before immediately. Here, the relative hydrolase activity of the preincubated sample at 40°C was regarded as 100%.

For determination of the thermostability and pH stability, samples were incubated in the reaction mixture as described above for 2 h. The retaining hydrolase activity was determined as mentioned above.

### Effects of different metal ions and protein inhibitors on hydrolase activity

To determine the effects of different metal ions on hydrolase activity, enzyme assay was performed in 0.05 M phosphate buffer (pH 6.5) with 50 mg·L^−1^ chlorpyrifos as a substrate and with various metal ions at a final concentration of 1.0 mM. The activity assayed in the absence of metal ions was defined as control. The metal ions tested include Mn^2+^, Zn^2+^, Cu^2+^, Mg^2+^, Fe^3+^, and Hg^2+^.

The effects of protein inhibitors (ethylenediamine tetraacetic acid (EDTA), glutathione, Tween 80, Triton X-100, SDS, *β*-mercaptoethanol, and 1,4-dithiothreitol (DTT) at a final concentration of 1.0 mM, respectively) on hydrolase activity were determined in the reaction mixture as described above. The purified enzyme was preincubated with the respective compound for 10 min at 40°C, followed by the standard enzyme assay as described above. The relative activity assayed in the absence of the protein inhibitors was regarded as 100%.

### Kinetic parameters determination

The effect of chlorpyrifos concentration, ranging from 1.14×10^−4^ to 5.71×10^−4^ mM, on enzyme activity was estimated under optimal assay conditions (40°C, pH 6.5 and 10 min). The kinetic parameters (Michaelis-Menten constant, *K*_m_, and maximal reaction velocity, *V*_max_) were determined by linear regression from double-reciprocal plots according to Lineweaver-Burk. The *K*_m_ and *V*_max_ were expressed in μM and μmol·min^−1^, respectively. The catalytic constant (*k*_cat_) was determined using the algorithm as expressed in Eq.(1).
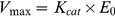
(1)where *E*_0_ is the initial concentration of enzyme, *V*_max_ is maximal reaction velocity, and *k*_cat_ is the catalytic constant (min^−1^), respectively.

### Substrate specificity determination

Substrate specificity against different organophosphorus insecticides (chlorpyrifos, parathion, methyl parathion, methamidophos, and malathion) was determined by measuring the hydrolase activity over a range of finial concentrations from 1.0 to 6.0 mM according to different pesticides. The measurement was prepared by the method of Liang et al. [32].

### Mass spectrometry for protein identification

The purified enzyme was electrophoretically transferred from a 12.5% SDS-polyacrylamide gel to a PDVF membrane (Millipore, USA). The region containing the chlorpyrifos hydrolase band was cut out, and the protein was sent to Shanghai GeneCore BioTechnologies Co., Ltd., China, for NH_2_-terminal sequencing. Sequencing was also performed using matrix-assisted laser desorption/ionization time-of-flight/time-of-flight mass spectrometer (MALDI-TOF-MS; Bremen, Germany) according to the method of Peng et al. [37].

### Analytical methods

Chlorpyrifos and other organophosphorus insecticides were analyzed on an Agilent 1100 HPLC (Agilent, USA) equipped with a Hypersil ODS2 C_18_ reversed phase column (4.6 nm×250 mm, 5 μm) with array detection from 190-400 nm (total scan). A modified method from Xu et al. [7] was used to determine the pesticide residues. In brief, samples were extracted using equal volume of acetone and dichloromethane (1∶1, *v/v*). After partitioning, clean-up, and concentration, residues dissolved in acetone was analyzed by HPLC. A mixture of methanol and water (70∶30, *v/v*) was used as the mobile phase at a flow rate of 1.0 mL·min^−1^ after acidification to pH 3 with concentrated phosphoric acid. The injection volumes were 10 μL. Recoveries of the organophosphorus insecticides added at concentrations of 1, 10, 50 mg/L, ranged from 87.0% to 103.5%. The linear range of the calibration curves was from 25 to 125 mg/L. The limits of detection (LOD) and quantification (LOQ) obtained during validation were 0.05 and 0.5 mg/L, respectively.

All of the experiments were carried out in triplicate, and the results were the means of three replicates. Standard deviations were also determined using Statistic Analysis System (SAS) software packages (Version 9.0).

## Results

### Purification of chlorpyrifos hydrolase

**Figure 1:**
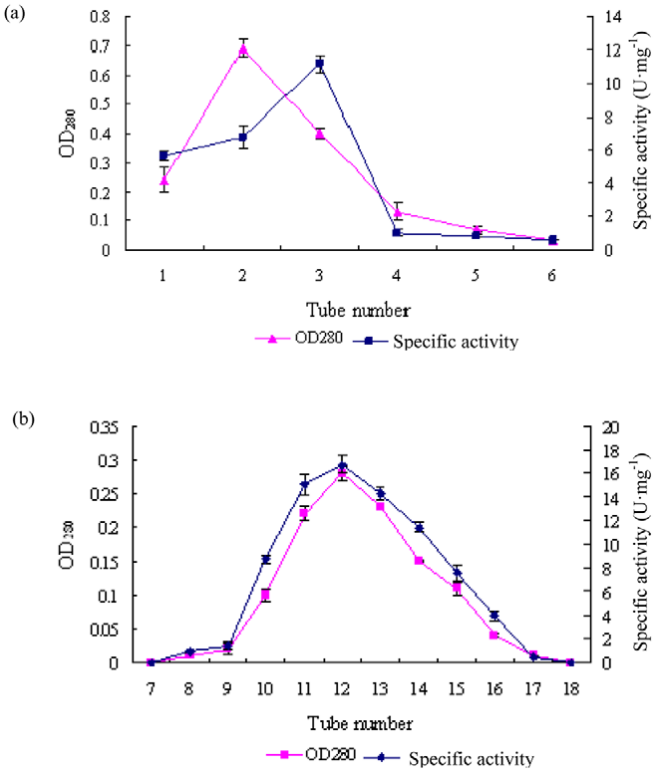
Elution profiles of chlorpyrifos hydrolase from *Cladosporium cladosporioides* Hu-01 on Sephacryl S-100 gel filtration (a) and DEAE Sepharose Fast Flow anion-exchange column (b).

The hydrolase activity increased with increase of cultivation time and reached peak of activity (51.1 U·mL^−1^) on the fifth day, then decreased. Hence, the collection time of the enzyme was determined for the purification. Chlorpyrifos hydrolase was purified from the cell extract of *C. cladosporioides* Hu-01 by ammonium sulfate precipitation, Sephacryl^TM^ S-100 gel filtration chromatography, and DEAE Sepharose Fast Flow anion-exchange chromatography. The result of the purification was shown in Fig. 1a and b and summarized in Table 1.

**Table 1:** Purification of chlorpyrifos hydrolase from *Cladosporium cladosporioides* Hu-01.

Purification steps	Total protein (mg)	Total activity (U)^a^	Specific activity (U·mg^−1^)	Purification (fold)	Yield (%)
Cell-free crude enzyme	544.8±26.8	256.6±10.2	0.5±0.1	1.0	100
Ammonium sulfate precipitation	323.9±9.9	207.3±9.5	0.6±0.1	1.4	80.8
Gel filtration chromatography	14.8±2.3	138.9±9.9	9.4±0.8	20.0	54.1
Anion-exchange chromatography	5.9±0.4	98.7±7.0	16.7±2.1	35.6	38.5

Note: ^a^ One unit of enzyme activity (U) was defined as the amount of enzyme that hydrolyzed 1 mmol of chlorpyrifos per minute. The data presented are means of three replicates with standard deviation, which is within 5% of the mean.

The elution profile of gel filtration chromatography on Sephacryl^TM^ S-100 exhibited two protein peaks (Fig. 1a). Then the two peaks of enzyme solution was subjected to DEAE Sepharose Fast Flow anion-exchange chromatography, the protein was eluted as a single peak with the specific activity (Fig. 1b), showing that pure chlorpyrifos-hydrolase was obtained. Therefore, the fractions were collected and concentrated by ultrafiltration. After the final purification step, the enzyme was purified 35.6-fold to a specific activity of 16.7 U·mg^−1^ protein from the cell with a yield of 38.5%.

**Figure 2:**
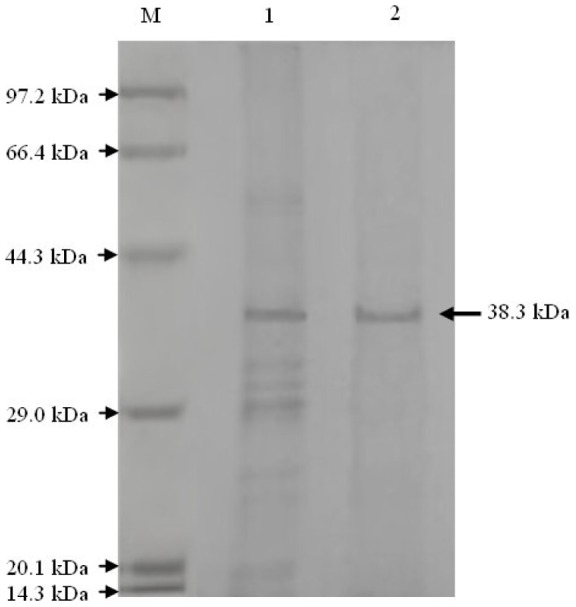
SDS-PAGE analysis on the purified enzyme. Lane 1: fractions from DEAE Sepharose Fast Flow anion-exchange; Lane 2: fractions from Sephacryl S-100 gel filtration; M: markers, 14.3-97.2 kDa.

The purified hydrolase indicated a single band on SDS-PAGE when stained with Coomassie brilliant blue R-250, with a molecular mass of around 38 kDa (Fig. 2). This result showed that the purified enzyme was purified to electrophoretic homogeneity. The relative molecular mass of native enzyme determined by gel filtration on a calibrated column of Sephacryl^TM^ S-100 was 38.3 kDa. These results revealed that the native chlorpyrifos hydrolase is a monomer. The pI value was estimated to be 5.2. Here, we designated the purified chlorpyrifos hydrolase as CPH.

### Effects of pH and temperature on hydrolase activity

**Figure 3:**
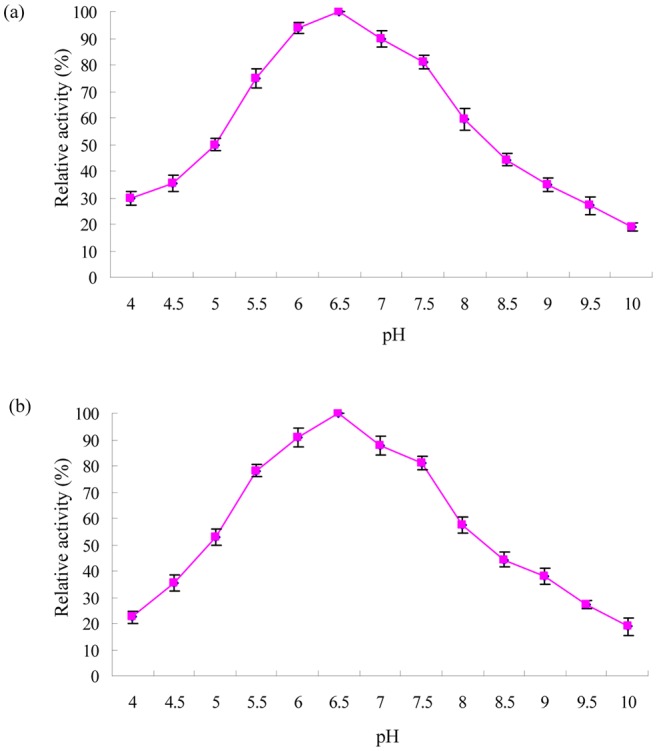
Effect of pH on enzyme activity (a) and stability (b) of chlorpyrifos hydrolase. Error bars represent the standard deviation of the means of three replicates.

The hydrolase activity was determined at various pH values in phosphate buffer with chlorpyrifos as a substrate. The results (Fig. 3a) showed that the optimal pH was observed to be 6.5. pH stability was measured via 2 h pre-incubation of the purified enzyme in the same buffer at different pH values ranging from 4.0 to 10.0 at 40°C. It can be seen from the results in Fig. 3b that the enzyme was very stable at pH 5.5 to 7.5, retaining more than 80% of the original activity after preincubation at that pH range for 2 h.

**Figure 4:**
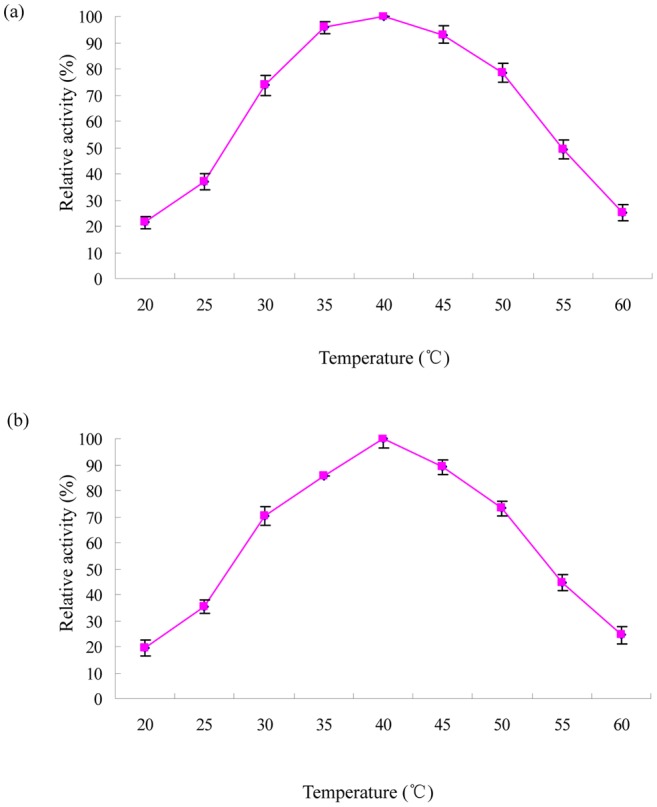
Effect of temperature on enzyme activity (a) and stability (b) of chlorpyrifos hydrolase. Error bars represent the standard deviation of the means of three replicates.

The hydrolase activity was most active at 40°C (Fig. 4a). Fig. 4a also indicates that the enzyme was inactivated rapidly at temperatures higher than 50°C and was almost inactivated at 60°C within 10 min. Thermostability of the purified enzyme was investigated by pre-incubating the enzyme in the same buffer as described above for 2 h and its residual activity was determined at different temperatures ranging from 20°C to 60°C at pH 6.5. As shown in Fig. 4b, the purified hydrolase was stable in the range of 30°C to 50°C and had 75% of its activity after treatment for 2 h.

### Effects of different metal ions and protein inhibitors on hydrolase activity

The effects of various chemicals on the enzyme activity were investigated by addition of the tested compounds into the reaction mixture at the final concentration (1.0 mM). The activity was then measured with chlorpyrifos as a substrate and expressed as a percentage of the activity obtained in the absence of the added compound (Table 2). Hg^2+^, Fe^3+^, DTT, *β*-mercaptoethanol, and SDS had strong inhibitory effect (65–95% inhibition); while Mn^2+^, Zn^2+^, Cu^2+^, Mg^2+^, and EDTA showed only slight inhibitory effect (5–10% inhibition). The presence of glutathione, Tween 80, and Triton X-100 resulted in approximately 15–25% inhibition of the enzymatic activity.

**Table 2:** Effects of various metal ions and protein inhibitors on chlorpyrifos hydrolase.

Metal ions	Relative activity (%)	Protein inhibitors	Relative activity (%)
Control	100±0.0	EDTA	95.4±1.2
Mn^2+^	96.2±0.7	Glutathione	87.5±3.4
Zn^2+^	95.2±2.4	Tween 80	81.6±4.1
Cu^2+^	94.7±1.2	Triton X-100	76.3±2.3
Mg^2+^	93.7±1.9	SDS	36.5±2.7
Fe^3+^	10.3±0.6	*β*-Mercaptoethanol	22.2±1.3
Hg^2+^	8.5±0.5	DTT	11.4±0.9

Note: The data presented are means of three replicates with standard deviation, which is within 5% of the mean.

### Substrate Specificity and kinetic analysis

**Figure 5:**
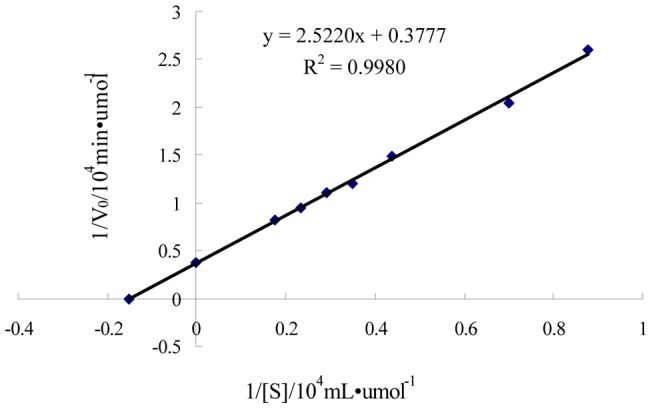
Lineweaver-Burk plot for *K*_m_ and *V*_max_ values of the purified enzyme in the presence of different concentrations of chlorpyrifos.

The substrate specificities of the purified enzyme were tested with various organophosphorus insecticides as the substrates. These pesticides included chlorpyrifos, parathion, methyl parathion, methamidophos, and malathion. Although the purified enzyme hydrolyzed all pesticides tested, the hydrolysis of chlorpyrifos was much higher than the hydrolysis of other organophosphorus insecticides. The hydrolase rates descending as follows: chlorpyrifos > parathion > methyl parathion > methamidophos > malathion. Chlorpyrifos was hydrolyzed rapidly with *K*_m_ and *V*_max_ values 6.7974 μM and 2.6473 μmol̇min^−1^, respectively, according to the Lineweaver-Burk plot (Fig. 5). The purified enzyme showed different hydrolysis rates against other organophosphorus insecticides with *K*_m_ values ranging from 9.8381 to 44.3833 μM (Table 3).

**Table 3:** Apparent kinetic constants for hydrolysis of different organophosphous pesticides.

Substrates	*V*_max_ (µmol·min^−1^)	*K*_m_ (µM)	*k*_cat_ (min^−1^)	*k*_cat_/*K*_m_ (µM^−1^·min^−1^)
Chlorpyrifos	2.6±0.06a	6.8±0.4e	1794.8±42.2a	264.0±13.4a
Parathion	2.3±0.15c	9.8±0.3d	1533.2±22.9b	155.8±7.0b
Methyl parathion	2.4±0.02b	12.5±1.4c	1630.5±45.5c	130.1±18.7c
Methamidophos	2.1±0.08d	31.8±1.7b	1403.3±20.5d	44.1±1.8d
Malathion	1.6±0.3e	44.4±2.1a	1103.5±22.8e	24.9±0.9e

Note: The data presented are means of three replicates with standard deviation, which is within 5% of the mean. Different letters in the same column indicate significant differences (*P*<0.05).

### Protein identification

**Figure 6:**
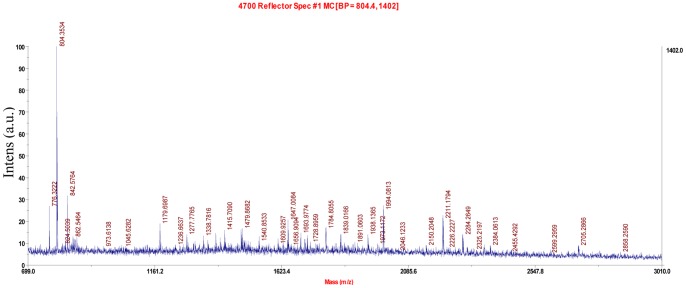
Matrix-assisted laser desorption/ionization time-of-flight/time-of-flight mass spectrometer (MALDI-TOF-MS) of the purified enzyme.

Fig. 6 shows the mass spectra of the purified hydrolase produced by *C. cladosporioides* Hu-01. One peptide fragment of the protein was measured by MALDI-TOF-MS and the amino acid sequence was MEPDGELSALTQGANS (Fig. S1). Database searches showed that the measured peptide shared no similarity with any reported organophosphate-hydrolyzing enzymes; however, it showed 30% identities to one fragment of the putative enzyme Q2G571_NOVAD from *Novosphingobium aromaticivorans strain DSM 12444*. These results suggested that the purified protein might be a novel chlorpyrifos hydrolyse.

## Discussion

Chlorpyrifos is one of the most popular insecticides used for pest control in agriculture since the 1960s, resulting in releasing of potentially toxic and persistent chemicals into the environment [27]. One of such chemicals is 3,5,6-trichloro-2-pyridinol (TCP), a breakdown metaboilte from chlorpyrifos and chlorpyrifos-methyl [1], [7], [38]. After their release, these pollutants can be transported through the atmosphere and water and, in many ways, find their way into sediments and soils, leading to widespread contamination [29]. Although several bacterial isolates has been reported to degrade chlorpyrifos [1], [7], [24]–[27], yet little information concerning the use of fungi for chlorpyrifos elimination is available. Fungus is an important member of microbes that are critical to the biogeochemical cycle and are responsible for the bulk of the degradation of hazardous materials in the biosphere [32]. However, the potential use for fungi or their enzymes in bioremediation of chlorpyrifos has not received the attention it deserves. To the best of our knowledge, this is the first report about bioremediation of chlorpyrifos using fungus from the genus *Cladosporium*.

In our studies, the screening of chlorpyrifos-degrading fungi by the method of enrichment procedure from soils contaminated with pesticides allowed us to select some potential isolates with a high survivability in the environment and maximal degrading activity towards chlorpyrifos. It was generally suggested that the conditions for environmental microorganism enrichment and screening are crucial in the selection of isolates with the desired degrading enzyme systems [7]. In most cases reported to date, the pesticide-degrading enzymes were isolated and purified from those microorganisms existing in contaminated soils, sediments, as well as industrial wastes [26], [27], [32], [36], [39]. Previous research showed that three degrading enzymes responsible for organophosphates degradation such as phosphotriesterase (PTE) or organophosphorus hydrolase (OPH), methyl parathion hydrolase (MPH), and organophosphorus acid anhydrolase (OPAA) have been isolated from various microorganisms and diverse geographic locations affected by pollutants [40]–[45]; however, the existing papers lack the information on the genetic and enzymatic aspects involved in the degradation of chlorpyrifos. This is the first report to our knowledge on the production, purification, and characterization of a chlorpyrifos hydrolase from fungus.

In the present study, the chlorpyrifos hydrolase was purified to apparent homogeneity from the cell extract of *C. cladosporioides* Hu-01 using ammoniumsulfate precipitation, gel filtration chromatography and anion-exchange chromatography. CPH has an apparent molecular mass of 38.3 kDa, which is smaller than those organophosphate-hydrolyzing enzymes from *Alteromonas* sp. JD 6.5 (60 kDa) and *Alteromonas undina* MG (53 kDa), but bigger than the methyl parathion hydrolase from *Pseudomonas* sp. WBC-3 (33.5 kDa) [41], [42], [44]. The enzyme appeared to be monomeric, this result was similar to the organophosphate hydrolase from *Pseudomonas diminuta* MG and *Agrobacterium radiobacter* P230 [40], [45]. The pI value of CPH was estimated to be 5.2, lower than that recorded for *Klebsiella* sp. ZD112 (8.6) and *Aspergillus niger* ZD11 (5.4) [32], [46]. The low pI indicates that the enzyme is rich in acidic amino acid residues [36].

The optimum pH of CPH (pH 6.5) was lower than those organophosphate hydrolases from *Pseudomonas* sp. WBC-3 (8.5), *Alteromonas undina* (8.0) and *Pseudomonas diminuta* MG (6.8) [40], [42], [44]. Furthermore, the chlorpyrifos hydrolase was very stable in the pH range of 5.5 to 7.5 (Fig. 3b). This result was in agreement with the observation that the stability of hydrolases is commonly between pH 5 and 8 [32], [36], [39]. The optimal temperature of CPH was 40°C, which was lower than that recorded for *Alteromonas undina* (55°C), but higher than that recorded for *Pseudomonas* sp. WBC-3 (30°C) [42], [44]. In addition, the purified enzyme was fairly stable in the temperature range of 30°C to 50°C (Fig. 4b). Hydrolases in general show optimal temperature mostly between 30°C and 50°C [32], [36], [39], [46].

The effects of various metal ions and potential inhibitors on the hydrolase activity were investigated. CPH apparently had no requirement for metal ions since chelating agent EDTA (1.0 mM) showed only slight inhibitory effect (approximately 5% inhibition) on CPH. This result contrasts with previous findings of Defrank and Cheng [42] who reported that the organophosphorous acid anhydrolase (OPAA) from *Alteromonas* sp. JD 6.5 was activated by Mg^2+^and Co^2+^, and Dumas et al. [40] and Horne et al. [45] who reported phosphotriesterase (PTE) from *Pseudomonas diminuta* MG and *Agrobacterium radiobacter* P230 contained two metal atoms (Fe^2+^ and Zn^2+^). However, the CPH was strongly inhibited by Hg^2+^, Fe^3+^, SDS, and thiol-modifying reagents such as DTT and *β*-mercaptoethanol, while other metal ions and protein inhibitors did not have a remarkable effect on the activity (Table 2). These results suggested that sulfhydryl groups may be involved in the catalytic center of the enzyme or in substrate binding and/or recognition. Similar results were observed in other hydrolases isolated from *Aspergillus niger* ZD11, *Sphingobium* sp. JZ-2 and *Sphingobium* sp. JZ-1 [32], [36], [39].

CPH not only hydrolyzed the organophosphorus insecticides containing a P-O bond such as chlorpyrifos, parathion and methyl parathion, but also degraded the organophosphates jointed by a P-S bond such as methamidophos and malathion, indicating that the purified enzyme has relatively broad specificity. However, the catalytic efficiency values (*k*_cat_/*K*_m_) of P-O bond pesticides were approximately 10-fold higher than P-S bond pesticides (Table 3), which suggested the hydrolase activity depended on the pesticide molecular structure. This observation did not quite agree with Dumas et al. [40] and Horne et al. [45] who reported the organophosphate hydrolase from *Pseudomonas diminuta* MG and *Agrobacterium radiobacter* P230 has very broad substrate range with high hydrolase activity. The apparent *K*_m_ and *V*_max_ values of CPH produced by *C. cladosporioides* Hu-01 for chlorpyrifos were 6.7974 μM and 2.6473 μmol·min^−1^, respectively. In contrast, *K*_m_ value of the PTE produced by *Pseudomonas diminuta* MG for paraoxon was 0.1 μM, *K*_m_ value of the MPH produced by *Plesiomonas* sp. M6 and *Pseudomonas* sp. WBC-3 for methyl parathion was 0.037 μM, and *K*_m_ value of the OPAA produced by *Alteromonas* sp. JD 6.5 for paraoxon was 14 μM, respectively [40], [41], [43], [44].

The NH_2_-terminal sequence containing the amino acids MEPDGELSALTQGANS showed 30% identities to one fragment of the putative enzyme Q2G571_NOVAD from *Novosphingobium aromaticivorans strain DSM 12444*. However, there was no homology with the previously described organophosphate-hydrolyzing enzymes or with other recognized hydrolases that have been isolated from microorganisms and insects [40]–[45]. Furthermore, the CPH described here differs from those previously available in at least one of the following aspects: molecular mass, pI, optimum pH and temperature. These results suggested that the CPH might be a novel hydrolyse, which shares no similarity with any reported organophosphate hydrolyses.

The accumulation and persistent use of organophosphorus pesticides worldwide is a growing global health issue that requires attention. Effective methods for disposal of these toxic compounds are needed to ensure that human and environmental health. Organophosphates-hydrolyzing enzymes are not only cost-effective but also they show good reaction efficiency with organophosphate substrates and provide an environmentally friendly solution to the problem of organophosphate compound detoxification [11]. Thus, it is critical to continue to search for and engineering of microbial enzymes from environmental isolates for rapid degradation of these compounds. In view of the resistance to many metal ions, leakage requirement for cofactors, relatively broad pH and temperature, and high activity against chlorpyrifos, the purified enzyme implied for development of a bioremediation strategy of chlorpyrifos-contaminated environments. However, further study is helpful before the application of the enzyme in situ, such as molecular knowledge on gene expression and regulatory mechanisms.

## Supporting Information

Figure S1 MS/MS analysis of the different peptides of the purified enzyme.
(TIF)
