# Supplementary figures and images for: Purification and Characterization of a Novel Chlorpyrifos Hydrolase from Cladosporium cladosporioides Hu-01
Source: PLoS One. 2012 Jun 5;7(6):e38137. doi: 10.1371/journal.pone.0038137 (PMC3367910; doi:10.1371/journal.pone.0038137)

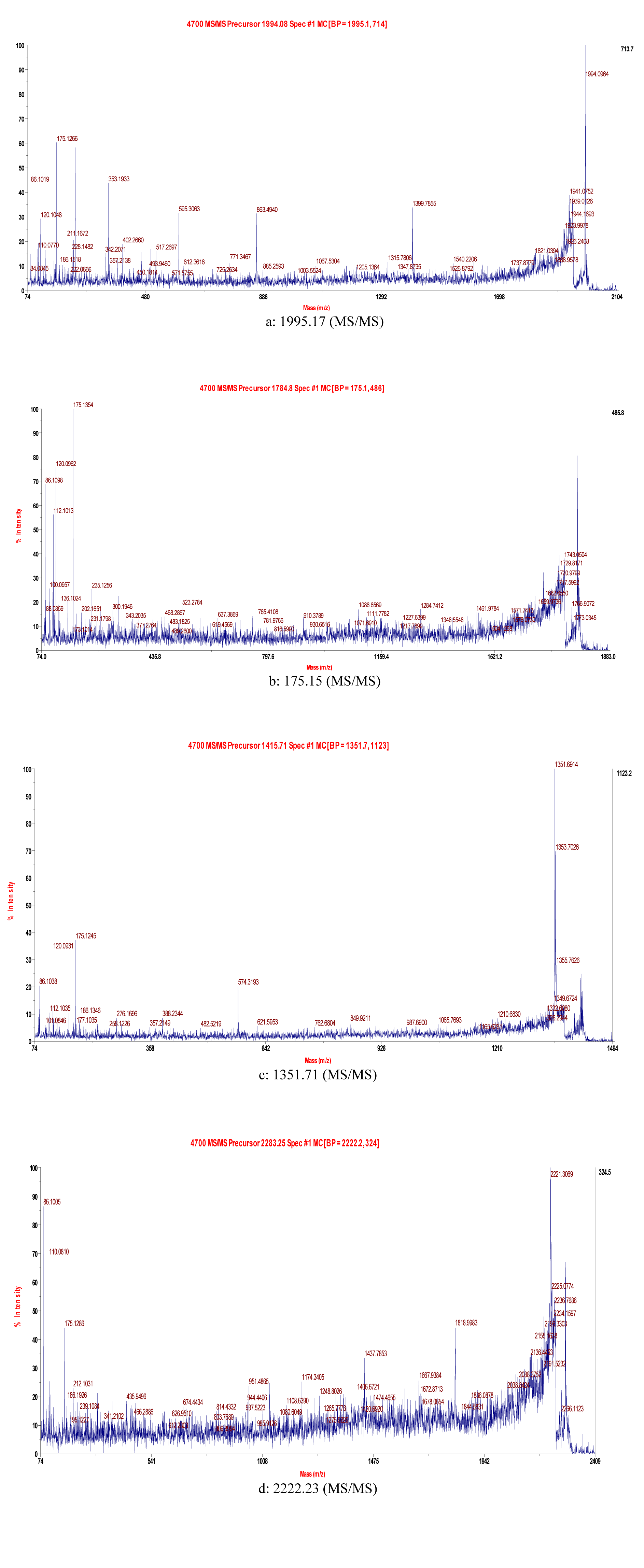

Supplement: Figure S1 — MS/MS analysis of the different peptides of the purified enzyme. (TIF) [file pone.0038137.s001.tif]
